# Supplementary material for: Supplement usage and doping attitudes in elite youth sports: The mediating role of dietary supplement acceptance
Source: PLoS One. 2024 Feb 1;19(2):e0297078. doi: 10.1371/journal.pone.0297078 (PMC10833512; doi:10.1371/journal.pone.0297078)

# Supplementary Material

## Mediation analysis with SEM

The following is a presentation of the computer syntax [M*plus*] for testing the half-longitudinal mediation model for the dietary supplement acceptance in the relationship between supplement use and doping attitudes.


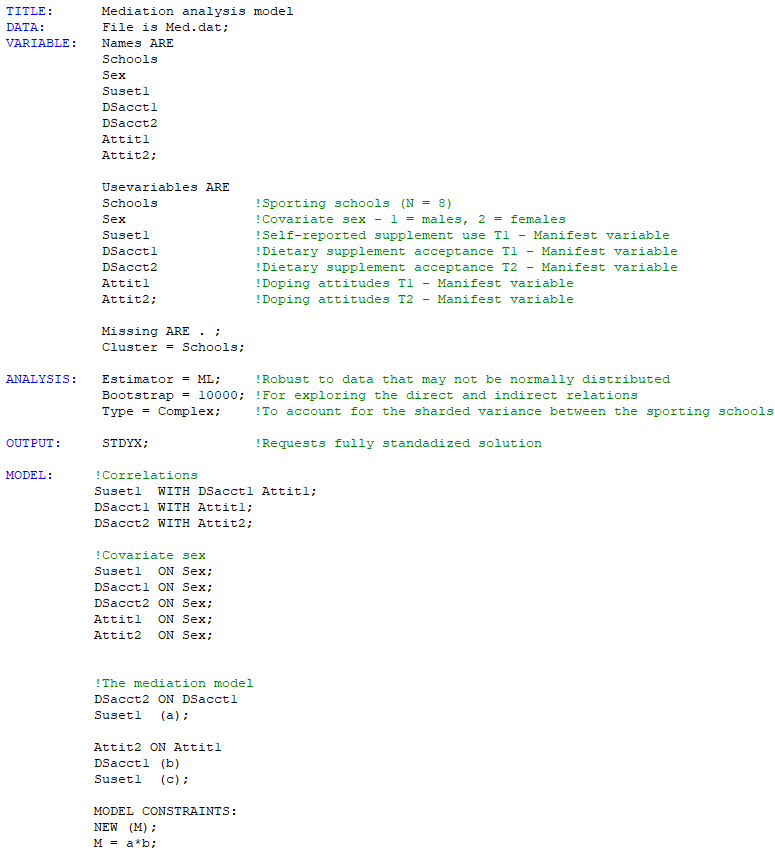

Supplement: S1 File — (DOCX) [file pone.0297078.s001.docx]
